# Determinants of green growth in developed and developing countries

**DOI:** 10.1007/s11356-021-13429-0

**Published:** 2021-03-22

**Authors:** Vincent Tawiah, Abdulrasheed Zakari, Festus Fatai Adedoyin

**Affiliations:** 1 DCU Business School, Dublin City University, Dublin, Ireland; 2 Center for Energy and Environmental Policy Research, School of Management and Economics, Beijing Institute of Technology, Beijing, China; 3 Alma Mater Europaea ECM, Maribor, Slovenia; 4 Department of Computing and Informatics, Bournemouth University, Poole, UK

**Keywords:** Energy consumption, Green growth, CO_2_ emissions, Environmental quality

## Abstract

Considering the need for environmental sustainability while ensuring economic growth and development by 2030, this study uses data on 123 developed and developing countries to examine factors that influence green growth. The empirical results show that economic development positively influences green growth. However, trade openness is detrimental to green growth. Regarding energy-related factors, we find energy consumption negatively affecting green growth, but renewable energy consumption significantly improves green growth. In further analysis, we find that the influence of these factors differs between developed and developing countries. The result implies that countries at a different development level will require different strategies in achieving the Sustainable Development Goals in 2030. The results are robust to alternative identification strategies such as the System Generalised Method of Movement, which accounts for potential endogeneity.

## Introduction

The continuous rise in temperature and its concomitant effect on livelihood has put sustainable development as the top priority in international discourse (IPCC 2018)^1^. Many nations have also continuously look for avenues to address climate change. The Paris Agreement and 2030 Sustainable Development Agenda have renewed the actions towards a better environment (Organisation for Economic Co-operation and Development—OECD 2020). Indeed, OECD (2020) suggests that Agenda 2030 for sustainable development is the norm for all countries, been developed or developing country. Despite these commitments by almost all countries, there are significant variations at which countries are moving towards environmentally sustainable economic development. For example, among OECD members, Australia and Belgium are experiencing a significant increase in green growth, but Portugal and Turkey are stagnant or decreasing in green growth.

Reflecting on the multi-facet nature of environmental issues and the fact that one size fit all strategy cannot solve these issues in this age of increasing economic activities, we examine the different set of factors that drive green growth. According to OECD (2020), green growth indicates whether economic growth is becoming greener with more efficient use of natural capital. The green growth indicator monitors progress towards a sustainable and greener economy (OECD 2020). Attaining green growth simply implies the use of natural assets towards economic growth in a sustainable manner. The goal is to move towards an economy that leads to human well-being and reduce inequalities among people in the long run, and not exposing future generations to environmental risk (OECD 2018).

We employ a fixed effect estimation technique on a large panel data of 123 countries over 18 years. The results show that economic development is positive and significantly associated with green growth, while trade openness negatively impacts green growth. We find no significant relationship between institutional quality and green growth. Although we find energy consumption to impair green growth, renewable energy consumption significantly increases green growth. Our results remain robust to different model specifications, including country effect and accounting for CO_2_ as an additional control variable. We also use the System Generalised Method of Movement to address any potential endogeneity issue. The results are also economically significant in explaining the variations of green growth across the world. For example, our results show that a 1% increase in trade openness, given its standard deviation, leads to 0.017 points decrease in green growth.

In further analyses, using sub-sampling techniques, we find interesting results between developed and developing countries. We find economic growth positive and significantly associated with green growth in developed countries but insignificant in developing countries. Next, while internationalization does not significantly impact green growth in developed countries, it is negatively and significantly associated with green growth in developing countries. Notwithstanding these contrasting findings, institutional quality and energy consumption remain similar for both developed and developing countries.

This study is timely as countries work towards achieving the Sustainable Development Goals, including high and progressive green growth by 2030. Our findings, which demonstrate the significant drivers of green growth, will enable policymakers to shape some of the country’s activities, leading to green growth. As evident in the results, countries at different development levels will require different strategies to achieve Sustainable Development Goals. For example, developing countries have to pay attention to foreign direct investment and trade they engage in to avoid harming the environment. Both developed and developing will also need to look closely at 'their institutional quality and regulations to boost green growth.

Our paper also makes an incremental contribution to the environmental literature by deviating from the traditional stream of carbon emission to a new area of improving its efficiency in using natural assets. More specifically, by focusing on green growth, we draw attention to factors that facilitate efficient and effective use of resources to achieve economic development and environmental sustainability.

The remainder of the paper is as follows. The next section presents a literature review on green energy determinants, capturing variables such as economic growth, foreign direct investment, foreign trade, and renewable energy, among others. Research methods, including data, description of variables, and econometric model, are presented in Section 3, while results are discussed in Section 4 with implications of results. The study concludes in Section 5 with vital policy recommendations.

## Review of prior studies

### Economic factors and the environment

Since the first Rio summit on environmental sustainability, the world falls short of numerous challenges, including the tense pressure to keep economic activities moving and the rising environmental degradation (The Washington Post 2017). Given these tense issues, green growth came to a place to bring these two issues together and address them (OECD 2011). In essence, green growth oversees economic growth and development while utilizing natural assets for the well-being of humanity. Despite this significant association between economic growth and green growth, there is scanty research on green growth. In light of the scarce literature on green growth, we present studies that may appear distinctively different but related to the environment and its consequence on green growth.

Alam and Kabir (2013) find that increase in economic growth sustains the environment by reducing carbon emissions. The author suggests joint pollution and eco-efficiency policies to enable environmental sustainability. Similarly, Rahman et al. (2020) posit the underline effect of dangerous emissions on economic growth to be approximately more than one digit, indicating that dangerous emissions alongside the population promote economic growth. However, the impact is insignificant because the trade openness causes a further decrease in economic growth. In another notion, investment in clean energy and advanced technologies is confirmed to effectively moderate pollution, which improves the economic growth of the host country (Muhammad and Khan 2019).

Furthermore, Mikayilov et al. (2018) confirm a long-run positive relationship between economic growth and a sustainable environment in Azerbaijan. These findings coined CO_2_ mitigating policies such as implementing the carbon price mechanism, public enlighten on the cause and danger of carbon dioxide emissions, and the use of less pollution-intensive technologies. Equally, economic growth could influence environmental performance regardless of the level of the education system. What matters the most is the mediating effect of education on the nation’s cultures and demographic density, which could prompt the influence of environmental performance (Peng and Lin 2009). Chang and Hao (2017) also confirm that environmental performance positively interacts with economic growth in OECD and non-OECD countries.

However, when output and consumption increase, we are most likely to observe cost imposed on the environment, which, by implication, increased the consumption of non-renewable resources to increase pollution levels. Ardakani and Seyedaliakbar (2019) and Xie and Liu (2019) sustain this claim and argue that economic growth below the turning point could cause more carbon emissions, but economic growth crosses the turning point, and then environmental quality is achieved or improve. Although economic growth enables inhabitants to maintain a higher life expectancy and increase enrolment to school, this impact may not hold volume to contain the pollution (Cracolici et al. 2010). Wang et al. (2019) find that investment and economic growth jointly contribute to environmental quality. The authors suggest emission mitigation policies that encompass the efficient use of energy, clean technology investment, and promotion of labour standards will cut down the rising emissions.

Similarly, Shahbaz et al. (2013) confirm economic growth as the major contributor to CO_2_ emission. The authors suggest a reduction at the cost of economic growth and financing the importation of environmentally friendly technologies. More so, the impact of economic growth may differ according to region. In China’s central and western region, Chen et al. (2019a, b) suggest that economic growth increases CO2 emissions when it is below a threshold level but reduces CO_2_ emission when the economic growth rises above a threshold. Adedoyin et al. (2020a, b) demonstrate that the BRICS economic’ CO_2_ emissions are aggravated by economic growth.

### Internalization and the environment

In the quest to grow the economic base, nations are pushed to embrace foreign investment and trade. Through foreign direct investment, a country can improve technological progress as well as promote human capital. Trade helps achieve an efficiency of production by allocating resources in areas where a country has a comparative advantage. However, the effect of both foreign direct investment and trade on the environment has not always been straightforward. Existing studies provide two contrasting findings on the impact of internationalization on the environment. Following the pollution haven hypothesis (Walter and Ugelow 1979), critics of internationalization argue that foreign investment and trade serve as a channel for transferring pollution-intensive operations from one country to another. Hence, foreign investment and trade are associated with the poor environmental quality of the host country while lowering pollution (Beradovic 2009). Haug and Ucal (2019) and Salahuddin et al. (2018) validate this claim and report that the underlying effect of foreign direct investment on carbon emission is positive and implies that an increase in FDI leads to a deteriorating environment. However, the long-run effect shows no significant relationship between FDI and carbon emissions (Ayamba et al. 2020). Meanwhile, trade is found to be asymmetrically correlated with carbon emission; a rise in export causes an increase in carbon emission, while a rise in import reduces carbon emission in Turkey. The reduction in CO_2_ emissions could have been caused by the transfer of highly emission-intensive production capacities from developed economies to low economies (Essandoh et al. 2020).

The pollution haven hypothesis is directly opposite to the pollution halo hypothesis, which states that internationalization facilitates the transfer of technology and acceptable practices from one country to another, particularly from developed to developing. Hence, foreign investment and trade improve the environmental quality of the host country (Birdsall and Wheeler 1993). There is a considerable amount of literature on which empirical evidence supports this hypothesis (Ayamba et al. 2019; Cole and Elliott 2003; Mihci et al. 2005; Pao and Tsai 2011; Romer 1993, Zhu et al. 2016). Mihci et al. (2005) argue that international investment and trade help fill the technology gap among countries. Shahbaz et al. (2019) argue that allowing free trading in and out of the country can improve environmental quality, but an increase in foreign investment is detrimental to a greener environment. The author suggests investment in technological innovation, capital stock, and the implementation of stringent environmental policies that will re-direct FDI towards green investment.

Furthermore, trade openness and FDI has pushed for healthy economic development, but not without contributing to pollution. However, Muhammad et al. (2020) provide evidence of the positive impact of import and export on the environment. Bopkin (2017) suggests that FDI can positively impact the environment if there are quality institutions to monitor foreign investors.

### Energy-related factors and the environment

High energy consumption is associated with low environmental quality (Bilgen 2014; Dincer 1998; Bailis et al. 2005; Fotis and Polemis 2018). Because energy consumption is inevitable in daily life, there is a strong argument for a different mix of energy sources of which renewable energy source has been considered the best alternative. Nonetheless, the empirical evidence on the impact of renewable energy on the environment remains very controversial, although it is perceived by many as a direct positive effect. Saidi and Omri (2020), for instance, claim that in order not to cause harm to the economy while promoting environmental quality, the private sector, such as industries, has to imbibe the use of nuclear and renewable energy during production. Equally, investment in human capital could cut-down emissions (Wang et al. 2020). Mendonca et al. (2020) confirm similar results in the 50 largest economies across the globe. According to Ikram et al. (2020), following the international standard’s issuance that specifies requirements for an effective environmental management system known as ISO 14001 has added a tally to factor helping to sustain the environment.

On the negative side, Jebli and Youssef (2017) argue that renewable energy consumption could raise CO_2_ emissions because combustible and wastes renewable energy are highly pollution-intensive energy sources. Hence, carbon emissions are likely to increase. Furthermore, renewable energy alone as an indicator may not effectively reduce the CO_2_ emissions until a fully renewable energy system is implemented, and then renewable energy will cut-down emissions (Pata 2018; Mitchell and Cleveland 1993). Kahouli (2018) reports that increasing renewable energy consumption could increase energy-intensive economic activities.

### Institutional quality and the environment.

The institutional quality, which includes the quality of laws and the strength of enforcement agents can either promote or retard green growth. A well-established institutional quality promotes economic activities while reducing carbon emissions (Salman et al. 2019). Specifically, political-institutional quality provides all the social, governance, and economic readiness to cut down carbon emissions (Sarkodie and Adams 2018). High institutional quality is more beneficial in improving a sustainable environment through the effect of trade than in low institutional quality (Ibrahim and Law 2016). Quality institutions also ensure that firms are complying with environmental regulations.

However, a strong enforcement environment with ever-changing regulations will likely hamper the growth of firms, especially foreign business flow. There could be less innovation and creativity towards improving the environment (Nguyen et al. 2018). It should be noted that the evidence on the negative impact of institutional quality is fragile. Therefore, Abid (2017) suggests that some parts of institutional quality, such as government effectiveness and democracy, deteriorate environmental quality while regulatory quality and the rule of law increase sustainability. Democratic institution paves the way for foreign investment at the expense of the environment. The fact is that foreign investors tried to avoid environmental laws; thus, they invest in the economy where such policies are not in place and thereby raise the pollution (Kinda 2011).

Previous studies have been conducted linking economic growth, development, renewable energy, foreign direct investment, and trade on the environment. However, the previous failed to account for the green growth determinant. Therefore, we fill this gap by investigating the determinants of green growth across a panel of 123 countries.

### Developed and developing countries

Given the difference between developed and developing countries regarding economic development and environmental degradation (De Angelis et al. 2019; Shahbaz et al. 2019), it is likely that their journey to green growth can be different. For example, whereas developing countries have a high pollution rate, developed countries are the largest contributors to CO_2_ emission but at a decreasing rate (De Angelis et al. 2019). Shahbaz et al. (2019) argue that the developed countries such as the US maintained policies within “scale effect” to reduce carbon emissions. This is done through investment in technological innovation, addressing capital consumption, and providing informed knowledge about trade liberalization has helped reduce CO_2_ emissions. Likewise, in Western Europe, CO_2_ emissions are well managed because of the enforcement of policies targeted at improving economic growth and environmental protection simultaneously (Paramati et al. 2017), which is not the case of Eastern Europe, which is made of developing countries. In Eastern Europe, CO_2_ emissions continue to surge beyond because the nation’s intent is directed towards tourism to grow its agenda for employment generation, income, and economic development without considering the environment (Paramati et al. 2017). This similar feature of most developing countries where there are weak institutions to enforce environmental laws.

In developing countries, Aye and Edoja (2017) found that economic growth has a negative impact on carbon emission in developing countries. However, Iwata et al. (2012) did not find any significant relationship between economic growth and carbon emission in developed countries. But the authors found a positive impact of energy consumption on carbon emission. Regarding the impact of foreign direct investment and trade, prior studies have found different results for developed and developing countries. Demena and Afesorgbor (2020) report that foreign direct investment reduces CO_2,_ but the result is stronger in developed countries than in developing countries. However, Khan et al. (2020) find FDI to negatively affect the environment in developing countries. Tang (2015) also reports a negative impact of FDI on the environment. Similarly, international trade has a negative impact on developing countries (Tawiah et al. 2021).

## Data and methods

### Data and sample selection

The sample selection begins with all 134 countries listed in the OECD database. We drop six countries that have missing data for more than 3 years on green growth. Next, we drop five countries with missing data from World Development Indicators. Our final sample covers a large panel of data of 123 developed and developing countries over 18 years (2000–2017). We begin in 2000 and end in 2017 because the OCED data on green growth is limited to only this period.

### Measurement of variables

#### Green growth

According to the OECD Statistic (2020), green growth is the measure of efficient use of natural capital. Green growth indicates whether economic growth is becoming greener. It captures aspects of production which are rarely quantified in economic models and accounting frameworks. Green growth is measured by the environmental and resource productivity of a country. The higher the value, the more the country’s economic growth is becoming greener. Data is sourced from OECD statistics.

#### Economic factors

These factors include the general economic activities of a country. We use GDP per capita to measure the effect of *economic development* on green growth. Similarly, we use annualized GDP growth to measure the impact of *economic growth*. Both variables are collected from World Development Indicators.

#### Internationalization factors

These determinants include trade openness and foreign direct investment. These factors are used to examine how foreign activities drive green growth. *Trade openness* is measured by the sum of imports and export as a percentage of GDP. *Foreign direct investment* is measured by the net inflow of foreign direct investment as a percentage of GDP. Following the pollution haven and pollution halo hypothesis, we expect the relationship between internationalization factors and green growth to go either way. Data on import, export, and foreign direct investment are sourced from World Development Indicators.

#### Institutional quality factors

Although there are different measurements of the institutional quality of a country including, the legal origin, bureaucratic quality, the Worldwide Governance Indicators (WGI) by Kaufmann and Kraay (2018) is the most widely used proxy for institutional quality (Elamer et al. 2020; Konara and Shirodkar 2018; Tunyi et al. 2020). Therefore, we use the six WGI indicators to measure the institutional quality of a country. These six indicators are government effectiveness, political stability, absence of violence, regulatory quality, the rule of law, voice and accountability, and control of corruption. According to Kaufman and Kraay (2018), these indicators cover institutional quality and governance’s core areas. Although each indicator covers different aspects of institutional quality, including all the six indicators in a single model as individual variables will create multicollinearity due to the high correlation (seen Table 9 of Appendix 2). To capture all the six indicators without the problem of multicollinearity, we follow prior studies such as Elamer et al., (2020), Konara and Shirodkar (2018), and Tunyi et al. (2020) and use the principal component analysis (PCA) technique to construct a single composite index from all the six indicators. The PCA statistics presented in Table 10 of Appendix 2 show that the first principal component explains about 86.65% of the variance among the components, whereas the other five components altogether capture less than 2% of the variance. Consequently, we use only the first principal component to predict the sample countries’ institutional quality index. We find our constructed index to be positively and significantly correlated with the average of the six indicators.

#### Energy-related factors

This group of determinants is related to the type and level of energy consumption in the country. It includes energy consumption measured by energy use and the type of energy consumption measured by *renewable energy*. Data on energy-related variables are collected from World Development Indicators.

#### Control factors

We control for other factors that are likely to influence green growth. We use a log *population* and *population growth* to control the effect of growing human activities on green growth. A large population exerts an enormous influence on the environment (Aller et al. 2015); therefore, we expect a positive relationship between population and green growth. We also use the *Forest area* as a percentage of the total land area to control the effect of a green environment on green growth. Arguably, countries with large forest areas are most likely to have access to a large pool of greener resources and increase green growth. *Natural resource* extraction has been found to have negatively affected the environment (Bokpin 2017; López 1994); hence we use resource rent to control for the effect of continuous extraction of natural resources on green growth.

### Pre-regression and econometric modelling

We perform different pre-regression tests to determine the appropriate estimate technique for the analysis. First, we test the correlation among the independent and control variables. Table 2 contains the result of the Pearson pairwise correlation matrix between the variables. The results show precursory evidence that selected variables are good for controlling for relevant factors that may influence domestic credit. However, none of the variables possesses any threat of multicollinearity, because all the coefficient is less than the standard threshold (Field 2000; Tabachnick and Fidell 2013). Next to minimize potential misspecification, we perform the Hausman test to choose between random effect and fixed effect estimation technique. The Hausman test results suggest that effects are not independent; hence, the use of the fixed effect is appropriate as opposed to the random effect.

Having established the data’s appropriateness for ordinary least square modelling, we fit our baseline as follows:

1 $$ {\mathrm{Green}\ \mathrm{grwoth}}_{\mathrm{it}}=a+{\beta}_1{\left(\mathrm{Determinants}\right)}_{\mathrm{it}}+{\beta}_2{\left(\mathrm{Control}\ \mathrm{variables}\right)}_{\mathrm{it}}+ $$

where *Determinants* take on *economic factors*, *internationalization factors*, *institutional quality factor*, *or energy-related factor* respective equations. *it* represent country and time respectively, and ε_it_ is the associated error. All variables are defined in Tables 1and 2.

**Table 1:** Description and sources of variables

Variable name	Measurement	Source
Green growth	Environmental and resources productivity	OECD Statistics
Economic determinants		
Economic development	Gross domestic product divided by population	World Development Indicators
Economic growth	Annualized growth in gross domestic product	World Development Indicators
Internationalization		
Trade openness	The sum of import and export as a percentage of gross domestic product	World Development Indicators
Foreign direct investment	The net inflow of foreign direct investment as a percentage of gross domestic product	World Development Indicators
Institutional quality		
Institutional quality	A composite index of the six Worldwide Governance Indicators	Worldwide Governance Indicators
Energy determinants		
Energy consumption	Energy use refers to the use of primary energy before transformation to other end-use fuels, which is equal to indigenous production plus imports and stock changes, minus exports and fuels supplied to ships and aircraft engaged in international transport	World Development Indicators
Renewable energy	Renewable energy consumption is the share of renewable energy in total final energy consumption	World Development Indicators
CO_2_ emission	Carbon dioxide emissions are those stemming from the burning of fossil fuels and the manufacture of cement. They include carbon dioxide produced during the consumption of solid, liquid, and gas fuels and gas flaring	World Development Indicators
Control variables		
Population	The estimated headcount of residents living in the country	World Development Indicators
Population growth	Annualized growth in population	World Development Indicators
Forest area	Forest area is land under natural or planted stands of trees of at least 5 m in situ, whether productive or not, and excludes tree stands in agricultural production systems and trees in urban parks and gardens. Measure as the percentage of the total land	World Development Indicators
Resource rent	Total natural resources rents are the sum of oil rents, natural gas rents, coal rents (hard and soft), mineral rents, and forest rents as a percentage of gross domestic product	World Development Indicators

**Table 2:** Pearson pairwise correlation matrix

Variables	1	2	3	4	5	6	7	9	10	12	13	14
Economic development	1											
Economic growth	−0.26	1										
Foreign direct investment	0.18	−0.04	1									
Trade openness	0.3	0.03	0.19	1								
Institutional quality	0.82	−0.22	0.19	0.32	1							
Energy consumption	0.66	−0.06	0.08	0.17	0.51	1						
Renewable energy	−0.57	0.11	−0.11	−0.26	−0.38	−0.37	1					
CO2 emissions	0.61	−0.02	0.08	0.16	0.41	0.9	−0.5	1				
Forest area	−0.01	−0.06	−0.06	−0.05	0.05	−0.12	0.3	−0.16	1			
Resource rent	−0.15	0.21	−0.06	−0.09	−0.4	0.14	0.01	0.24	−0.09	1		
Population	−0.26	0.08	−0.13	−0.42	−0.3	−0.31	0.13	−0.23	−0.06	−0.01	1	
Population growth	−0.12	0.18	−0.03	0	−0.23	0.22	0.16	0.27	−0.2	0.47	−0.02	1

**Table 3:** Descriptive statistics

Variables	*N*	Mean	P25	Median	P95	SD
Green growth	2214	5.977	3.343	4.963	13.26	4.058
Economic development	2210	8.645	7.474	8.612	10.88	1.504
Economic growth	2214	3.930	1.937	3.841	10.000	4.214
Trade openness	2214	86.34	55.62	77.60	163.8	53.89
Foreign direct investment	2110	3.225	0.0511	0.523	11.94	17.37
Institutional quality	2214	2.85e-09	−1.754	−0.462	3.998	2.280
Energy consumption	1862	2.670	650.4	1.615	8.512	3.055
Renewable energy	1968	28.28	5.658	19.13	86.22	27.49
Population	2214	16.30	15.24	16.17	18.89	1.661
Population growth	2214	1.357	0.414	1.159	3.634	1.672
Forest area	2091	30.76	11.03	31.13	68.41	22.15
Natural resources	2210	7.887	0.530	2.387	35.75	11.81
CO_2_ emissions	2081	5.941	1.151	3.808	19.64	7.306

**Table 4:** Main results

	(1)	(2)	(3)	(4)	(5)
Variables	Economic	Internationalization	Institutional quality	Energy	All factors
Economic development	0.282** (0.139)				0.461*** (0.151)
Economic growth	−0.000394 (0.00895)				−0.00219 (0.00854)
Trade openness		−0.00196* (0.00103)			−0.00430** (0.00217)
Foreign direct investment		−0.00355 (0.01860)			−0.00208 (0.00175)
Institutional quality			0.00187 (0.0132)		0.00108 (0.0116)
Energy consumption				−0.000337*** (5.63e-05)	−0.000333*** (5.12e-05)
Renewable energy				0.117*** (0.00862)	0.117*** (0.00818)
Population	−3.761*** (0.366)	−3.867*** (0.336)	−3.850*** (0.363)	−3.250*** (0.412)	−2.984*** (0.374)
Population growth	−0.0206 (0.0349)	−0.0121 (0.0317)	−0.0120 (0.0345)	−0.00990 (0.0341)	−0.0217 (0.0306)
Forest area	0.0488* (0.0250)	0.0877*** (0.0240)	0.0484* (0.0249)	−0.0575** (0.0284)	0.000734 (0.0266)
Natural resources	−0.0261*** (0.00827)	−0.0252*** (0.00814)	−0.0269*** (0.00815)	−0.0513*** (0.00942)	−0.0551*** (0.00933)
Constant	59.72*** (5.860)	62.59*** (5.175)	63.32*** (5.587)	53.12*** (6.392)	44.17*** (6.095)
Country effect	Yes	Yes	Yes	Yes	Yes
Observations	2087	1990	2087	1858	1767
*R*-squared	0.901	0.912	0.901	0.917	0.931
Number of countries	123	123	123	123	123

Standard errors in parentheses

***p<0.01, **p<0.05, *p<0.1

**Table 5:** Economic significance

Variables	Economic	Internationalization	Institutional quality	Energy	All factors
Economic development	0.07096				0.11600
Economic growth	NA				NA
Trade openness		−0.01767			−0.03877
Foreign direct investment		NA			NA
Institutional quality			NA		NA
Energy consumption				−0.17225	−0.17020
Renewable energy				0.53812	0.53812

Economic significance is calculated for only variables that are significant using the following formula

$$ \mathrm{Economic}\ \mathrm{significance}=\frac{\left(\mathrm{Cofficient}\ast \mathrm{standard}\ \mathrm{deviation}\ \right)}{\mathrm{Mean}\ \mathrm{of}\ \mathrm{green}\ \mathrm{growth}} $$

**Table 6:** Developed and developing countries

	Developed					Developing				
	(1)	(2)	(3)	(4)	(5)	(6)	(7)	(8)	(9)	(10)
Variables	Economic	Internationalization	Institutional quality	Energy	All factors	Economic	Internationalization	Institutional quality	Energy	All factors
Economic development	0.302* (0.160)				0.551*** (0.147)	0.832*** (0.178)				0.313** (0.103)
Economic growth	0.0375*** (0.00938)				0.0384*** (0.00801)	−0.00299 (0.0109)				−0.00158 (0.0110)
Trade openness		0.000503 (0.00171)			−0.000817 (0.00160)		−0.0120*** (0.00267)			−0.0102*** (0.00303)
Foreign direct investment		−0.000527 (0.00117)			−0.000614 (0.00100)		−0.0128*** (0.00295)			−0.00601** (0.00292)
Institutional quality			0.00254 (0.0111)		−0.00183 (0.00942)			0.00676 (0.0169)		0.000682 (0.0153)
Energy consumption				−0.0002*** (4.98e-05)	−0.0001*** (5.35e-05)				−0.00020** (8.14e-05)	−0.000131* (7.12e-05)
Renewable energy				0.101*** (0.0119)	0.113*** (0.0118)				0.107*** (0.0112)	0.0923*** (0.0102)
Constant	6.515 (4.248)	13.74*** (3.703)	10.42*** (3.674)	14.49*** (5.065)	5.847 (5.163)	106.3*** (9.692)	127.0*** (8.505)	119.6*** (9.294)	105.5*** (11.13)	105.0*** (10.92)
Country effect	Yes	Yes	Yes	Yes	Yes	Yes	Yes	Yes	Yes	Yes
Observations	578	561	578	536	521	1509	1429	1509	1322	1246
*R*-squared	0.943	0.943	0.941	0.956	0.961	0.905	0.919	0.904	0.914	0.931
Number of countries	34	34	34	34	34	89	89	89	89	89

Standard errors in parentheses

***p<0.01, **p<0.05, *p<0.1

**Table 7:** Accounting for CO2 emissions

	(1)	(2)	(3)	(4)
Variables	Full sample	Full sample	Developed	Developing
CO_2_ emissions	−0.224*** (0.0211)	−0.104*** (0.0259)	−0.00804 (0.0178)	−0.0148 (0.0455)
Economic development		0.526*** (0.151)	0.549*** (0.148)	0.328 (0.208)
Economic growth		−0.000337 (0.00851)	0.0385*** (0.00801)	−0.00156 (0.0110)
Trade openness		−0.00510** (0.00217)	−0.000904 (0.00161)	−0.0102*** (0.00303)
Foreign direct investment		−0.00195 (0.00174)	−0.000603 (0.00100)	−0.00596** (0.00292)
Institutional quality		0.00151 (0.0115)	−0.000146 (0.00949)	0.000629 (0.0153)
Energy consumption		−0.000192*** (6.19e-05)	−0.000163*** (5.79e-05)	−0.000116*** (8.47e-06)
Renewable energy		0.108*** (0.00846)	0.113*** (0.0122)	0.0916*** (0.0105)
Population	−5.438*** (0.385)	−3.596*** (0.402)	−0.719** (0.333)	−6.791*** (0.677)
Population growth	−0.00333 (0.0337)	−0.0240 (0.0305)	0.00293 (0.0176)	−0.0385 (0.0567)
Forest area	0.0147 (0.0246)	−0.00585 (0.0266)	0.109** (0.0532)	−0.0554* (0.0315)
Natural resources	−0.0271*** (0.00794)	−0.0524*** (0.00932)	−0.0225* (0.0123)	−0.0595*** (0.0113)
Constant	88.26*** (5.935)	53.38*** (6.488)	7.243 (6.229)	104.8*** (10.94)
Country effect	Yes	Yes	Yes	Yes
Observations	2077	1765	519	1246
*R*-squared	0.906	0.932	0.961	0.931
Number of countries	123	123	123	123

Standard errors in parentheses

***p<0.01, **p<0.05, *p<0.1

**Table 8:** Endogeneity results

	(1)	(2)	(3)
Variables	Full sample	Developed	Developing
Lagged green growth	−0.0389** (0.0157)	−0.111*** (0.0190)	−0.0225 (0.0210)
Economic development	0.685*** (0.0694)	1.470*** (0.116)	0.327*** (0.112)
Economic growth	0.0196 (0.0174)	0.0492*** (0.0182)	0.0319 (0.0236)
Trade openness	0.00504*** (0.00132)	0.0111*** (0.00108)	−0.00533** (0.00230)
Foreign direct investment	0.00329 (0.00365)	−0.00160 (0.00242)	0.00306 (0.00713)
Institutional quality	0.00163 (0.0283)	−0.00103 (0.0283)	0.0109 (0.0398)
Energy consumption	−0.000321*** (2.97e-05)	−0.000316*** (3.44e-05)	−0.000269*** (4.45e-05)
Renewable energy	0.112*** (0.00313)	0.0909*** (0.00579)	0.104*** (0.00418)
Population	0.0500 (0.0438)	0.310*** (0.0537)	−0.171*** (0.0620)
Population growth	0.266*** (0.0430)	0.0926** (0.0384)	0.277*** (0.0719)
Forest area	0.0139*** (0.00323)	−0.00719* (0.00373)	0.0232*** (0.00442)
Natural resources	0.0181*** (0.00578)	0.00260 (0.00842)	0.0227*** (0.00765)
Constant	−4.359*** (1.002)	−15.57*** (1.285)	2.567* (1.536)
Observations	1752	521	1231
Number of countries	123	34	89

Standard errors in parentheses

***p<0.01, **p<0.05, *p<0.1

## Results, discussion, and implications for policy

### Descriptive statistics

The descriptive statistics, including the mean, 25^th^ percentile, median 95^th^ percentile, and the standard deviation of all the variables, are presented in Table 3. The mean of *green growth* is 5.977, with a large standard deviation of 4.058, indicating different stages of green growth among the sample size. The mean of *economic growth* of 3.930, which is lower than the mean of green growth, shows that environmental and resource productivity increases even when general economic growth is low. We observe large variations across almost all the independent variables, suggesting that these variables could explain the green growth variations. The number of observations (N) is not the same for all variables, resulting in unbalanced data in the estimations.

### Main results

The fixed effect ordinary least square estimations are presented in Table 4. To mitigate potential bias results, the country-fixed effect is included in all the models. The results of economic determinants are presented in column 1. The economic development proxy coefficient by GDP per capita is positive and significant at 1%, indicating a positive relationship between economic development and green growth. However, the coefficient of economic growth is not significant. Therefore, our results in column 1 imply that high economic development promotes sustainable environmental and economic growth. The results are consistent with the assumption that countries with high GDP per capita have the resources to provide incentives towards a greener environment.

In column 2, we regress the internationalization factors, namely, trade openness and foreign direct investment on green growth. The results show that trade openness has a negative and significant association with green growth, which is in line with the findings of Alola et al. (2019a, b), whereas foreign direct investment also has a negative but insignificant relationship. These results suggest that countries that engage in more international business are more likely to experience a decline in green growth. The result is consistent with the pollution haven hypothesis, which states that the flow of international business deteriorates the environmental quality of the host country because of the transfer of pollution-intensive operations from one country to the other.

As presented in column 3 of Table 4, the result of institutional quality is insignificant, suggesting that the level of institutional quality is not a significant determinant of green growth. This is probably because most countries still do not have specific regulations on the sustainable use of natural assets. This is because most regulations on environmental protection are viewed to be counterproductive to economic growth and development (De Angelis et al. 2019; Wolde-Rufael and Weldemeskel 2020)

Column 4 contains the results of energy consumption as a determinant of green growth. As expected, high energy consumption is associated with a decrease in green growth. The coefficient of *energy consumption* (−0.000337***) is negative and highly significant, indicating an inverse relationship between the use of energy and green growth. However, the positive coefficient of *renewable energy* (0.117***) at a 1% significance level suggests that the type of energy matters in attaining green growth. More specifically, the results show that renewable energy consumption leads to an increase in green growth. Arguably, renewable energy sources make efficient and effective use of natural assets in general production and consumption than any other energy source.

Finally, in column 5, we combine all the factors, including the control variables, into a single equation. The results are not qualitatively different from the individual equations, confirming the results presented in columns 1–4. We find that both economic development and trade openness are larger in the combined equation than the individual equations.

Collectively, the results presented in Table 4 show that internationalization factors such as trade openness derive down green growth, while economic development is associated with increased green growth. Energy consumption has a negative impact on green growth; however, renewable energy consumption increases green growth.

The results of most control variables meet the standard assumption. For example, the population and extraction of natural resources are associated with a decrease in green growth. In contrast, countries with large forest areas experience green growth. The large and consistent *R*-squared across all the models signals how well the selected variables explain green growth variations.

To check the sanity of our results, we present the economic significance of the determinants that are statistically significant in Table 4. The results in Table 5 show that all the statistically significant variables are also economically significant. For instance, the results in column 1 show that an increase in economic development leads to an increase in green growth by 0.0709. However, an increase in trade openness leads to a decrease in green growth by 0.017. Similar energy consumption decreases green growth by 0.172, but renewable energy consumption increases green growth by 0.538. The economic significance is calculated as (coefficient*standard deviation)/mean of green growth.

### Developed and developing countries

Given that our sample size is large, it is likely that our results could be driven by a particular set of countries, especially developed or developing countries. The level of environmental pollution differs between developed and developing. Whereas developing countries have a high pollution rate, developed countries are the largest contributors to CO_2_ emission but at a decreasing rate (De Angelis et al. 2019). Furthermore, developed countries have high economic development but slow economic growth compared to the high economic growth but low economic development of developing countries. What is more, our large sample size contains 89 developing countries and 34 developed countries; hence, developing countries can drive our main results. Consequently, in this section, we use sub-sampling estimation techniques to check whether the results on green growth determinants differ between developed and developing countries.

The results which are presented in Table 6 reveal interesting and contrasting findings between developed countries and developing countries. First, on the economic determinants, economic development is positive and significant for both developed and developing countries, which is consistent with the full sample in Table 4. However, economic growth is positive and significant for developed countries (Alola et al. 2019a, b) but is insignificant for developing countries. This contrasting result suggests that developing countries that are growing fast are over-utilizing their natural assets, but developed countries experiencing fast economic growth are efficient in managing their natural assets towards green growth. This is consistent with Shahbaz et al. (2019) and Akadiri et al. (2019) findings that developed countries incorporate efficient technical process in their growth to reduce carbon emissions.

Second, on internationalization factors, the results show that both trade openness and foreign direct investment do not have a significant relationship with green growth in developed countries. In sharp contrast, the coefficient of both trade openness and foreign direct investment is negative and significant in column 7, indicating that internationalization is detrimental to green growth in developing countries. This negative result leans support to the pollution haven hypothesis that most international businesses in developing countries are pollution-intensive, as such foreign direct investment and trade reduce green growth in developing. These results are consistent with Khan et al. (2020) and Tawiah et al. (2021).

Third the results of institutional quality and energy-related factors are similar for both developed and developing countries and consistent with the main results as presented in Table 4. There is an inverse relationship between energy consumption and green growth, but the consumption of renewable energy increases green growth in all countries regardless of whether it is classified as developed or developing.

In sum, the results in Table 6 highlight that the economic and internationalization determinants of green growth differ between developed and developing countries, but the relationship between green growth and energy consumption is similar for both sets of countries.

### Accounting for the level of CO_2_ emission

Although green growth may appear synonymous with CO_2_, they are quite different. CO_2_ emission measures the environmental footprint, while green growth measures the action a country is taken to achieve growth and development, which is environmentally and economically sustainable. In other words, green growth involves reducing environmental footprint. Therefore, it is more likely that countries with high CO2 emissions have more incentive to engage in green growth due to the inherent obligation of solving the environmental problem of which they are the contributors. Arguably high CO_2_-emitting countries like Australia, China, the USA, and India have more incentives to increase green growth than countries like Portugal, Spain, Lao, and Angola, which have low emissions. Consequently, in this section, we test if the CO2 emission of the country drives our results.

The results are presented in Table 7. We begin by establishing the relationship between CO2 emission and green growth without the determinants but including the control variables. The results presented in column 1 show that CO_2_ emission has a negative effect on green growth. Next, we include all the determinants for the full sample in column 2, developed countries in column 3, and developing countries in column 4. The coefficient of CO_2_ emission remains negative and significant for the full sample but insignificant for both developed and developing countries, suggesting that CO2 emission is less likely to be a significant determinant of green growth based on country classification. The other determinants remain qualitatively similar to the main results, which confirm our estimation’s robustness after accounting for the level of CO_2_ emission.

### Robustness check

In this section, we use the System Generalized Method of Movement (S-GMM) to test the robustness of the results to potential endogeneity problems. The S-GMM estimator can control for the presence of unobserved country-specific effects. The S-GMM estimation technique also has the advantage of controlling for or a simultaneity bias caused by the potential endogeneity of the explanatory variables (Arellano and Bond 1991).

The results of the S-GMM estimations are presented in Table 8. The full sample results are presented in column 1, whereas those of developed and developing countries are presented in columns 2 and 3, respectively. The results are qualitatively similar to those in the main result of Tables 4 and 6; hence our results are not sensitive to potential endogeneity problems.

## Conclusion and policy implications

This study investigates the determinants of green growth in 123 developing and developed countries listed in the OECD database between 2000 and 2017. Findings from the study show that high economic development promotes sustainable development and economic growth, which is in line with the assumption that countries with high GDP per capita possess adequate resources to support green growth incentives. This implies the need to adopt economic development policies, which leads to increased GDP since it is a determinant of achieving green growth goals and sustainability goals.

On the other hand, trade openness was found to exert a negative and significant relationship with green growth and foreign direct investments having a negative but insignificant relationship with green growth. This implies that increased international activities such as trade and foreign investments may deter or slow countries’ green goals. Therefore, it is important to ensure prompt restrictions and monitoring of foreign investments and movements to ensure the economic growth effect of FDI and trade is accompanied by environmental quality. The government and regulatory agencies of developed and particularly developing countries should be deliberate in their sustainability goals to ensure green growth. Meanwhile, the institutional quality is an insignificant determinant of green growth, i.e., considering the role of government and institutional policies in the success of economic goals such as green growth, the quality of environmental institutions in determining the green growth achievement is insignificant. Findings further show that high energy consumption leads to a decrease in green growth; however, renewable energy use improves green growth. This shows that despite energy consumption deterring green growth, renewable energy dominating the energy mix contributes positively to green growth and environmental sustainability.

In additional analyses, using the sub-sampling technique, we find that our results are sensitive to countries been classified as developed or developing countries. For instance, the economic development of green growth for both developed and developing countries is positive and significant. In contrast, economic growth is positive and significant for developed countries but insignificant for developing countries. On the other hand, the internationalization determinants of green growth, trade openness, and FDI relationship with green growth are insignificant for developed countries. The effect of trade openness and FDI on developing countries is negative and significant, causing FDI and trade openness to be detrimental to green growth. However, the effect of institutional quality in determining green growth increase is insignificant for both developed and developing countries.

Given our findings, we recommend maintaining a high level of GDP per capita to attain green growth through the purchase of resources tantamount to green growth. According to the IMF report (2019), trade and technology are famous for promoting GDP growth and GDP per capita. Hence, countries can gear up their policies towards the promotion of GDP per capita through technological innovations.

Second, government and policymakers should carefully choose international business policies because the foreign direct investment is often not targeted towards green growth. For instance, international business contract host countries often are at a disadvantage because of the transfer of pollution-intensive operations from one country to the other, thereby creating pollution. Against this background, we suggest the employment of minimal foreign investment with strict supervision in order not to give room for exploitation by foreign counterparts.

Third, renewable energy sources make efficient and effective use of natural assets in general production and consumption than any other energy source. As such, it is more likely to promote green growth than other sources of energy. For these reasons, we encourage governments and policymakers to promote renewable energy by offering grants and loans to investors in those areas. Also, a tax holiday could be another option to promote investment in renewable energy. With that, more users are likely to increase, thereby lowing the level of CO2 emissions and, consequently, achieving green growth.

Our results are not only statistically significant; they are also economically significant in explaining the variations in green growth across the world. Further, our results are robust to alternative identification, including System Generalised Method of Movements, which mitigate potential endogeneity.

## Data Availability

The data is accessible from the author upon request.

## Appendix 1. List of countries

**Table:**

Albania	Canada	Georgia	Kuwait	Nigeria	Spain
Algeria	Chile	Germany	Kyrgyz Republic	Norway	Sri Lanka
Angola	China	Ghana	Latvia	Oman	Suriname
Argentina	Colombia	Greece	Lebanon	Pakistan	Sweden
Armenia	Congo, Dem. Rep.	Guatemala	Lithuania	Panama	Switzerland
Australia	Congo, Rep.	Honduras	Luxembourg	Paraguay	Tanzania
Austria	Costa Rica	Hungary	Malaysia	Peru	Thailand
Azerbaijan	Cote d'Ivoire	Iceland	Malta	Philippines	Togo
Bahrain	Croatia	India	Mauritius	Poland	Trinidad and Tobago
Bangladesh	Cyprus	Indonesia	Mexico	Portugal	Tunisia
Belarus	Czech Republic	Iran, Islamic Rep.	Moldova	Qatar	Ukraine
Belgium	Denmark	Iraq	Mongolia	Romania	United Arab Emirates
Benin	Dominica	Ireland	Morocco	Russian Federation	UK
Bolivia	Ecuador	Israel	Mozambique	Saudi Arabia	USA
Bosnia and Herzegovina	Egypt, Arab Rep.	Italy	Myanmar	Senegal	Uruguay
Botswana	El Salvador	Jamaica	Namibia	Serbia	Uzbekistan
Brazil	Estonia	Japan	Netherlands	Singapore	Vietnam
Brunei Darussalam	Ethiopia	Jordan	New Zealand	Slovak Republic	Yemen, Rep.
Bulgaria	Finland	Kazakhstan	Nicaragua	Slovenia	Zambia
Cambodia	France	Kenya	Niger	South Africa	Zimbabwe
Cameroon	Gabon	Korea, Rep.			

## Appendix 2. PCA statistics

**Table 9:** Correlation matrix between WGI

Variables	1	2	3	4	5	6
1. Corruption	1					
2. Government effectiveness	0.9503	1				
3. Political stability	0.7616	0.7465	1			
4. Regulatory quality	0.9051	0.939	0.7377	1		
5. Rule of law	0.963	0.9613	0.7815	0.937	1	
6. Voice and accountability	0.7841	0.784	0.6628	0.8178	0.8117	1

**Table 10:** Eigenvalues

Component	Eigenvalue	Difference	Proportion	Cumulative
Comp1	5.19882	4.84294	0.8665	0.8665
Comp2	0.355875	0.074561	0.0593	0.9258
Comp3	0.281315	0.187736	0.0469	0.9727
Comp4	0.093579	0.053709	0.0156	0.9883
Comp5	0.03987	0.009324	0.0066	0.9949
Comp6	0.030546	.	0.0051	1

## Footnotes

1 All over the world, many countries have recorded greater warming in their regions, with about half of the world populace experiencing warm temperatures of above 1.5°C in at least one period or the other (IPCC 2018).
